# Multi-functional nano-adhesive releasing therapeutic ions for MMP-deactivation and remineralization

**DOI:** 10.1038/s41598-018-23939-6

**Published:** 2018-04-04

**Authors:** Soo-Kyung Jun, Sun-A. Yang, You-Jin Kim, Ahmed El-Fiqi, Nandin Mandakhbayar, Duck-Su Kim, Jiyeon Roh, Salvatore Sauro, Hae-Won Kim, Jung-Hwan Lee, Hae-Hyoung Lee

**Affiliations:** 1 Department of Dental Hygiene, Kyungdong University, Wonju 26495, South Korea; 2 Department of Biomaterials Science, School of Dentistry, Dankook University, Cheonan 31116, South Korea; 3 Institute of Tissue Regeneration Engineering (ITREN), Dankook University, Cheonan, 31116 South Korea; 4 Lithuanian University of health sciences, Kaunas, 44307 Lithuania; 5 Glass Research Department, National Research centre, Cairo, 12622 Egypt; 6 Department of Conservative Dentistry, School of Dentistry, Kyung Hee University, 02447 Seoul, South Korea; 7 Department of Dental Hygiene, Yonsei University Wonju College of Medicine, Wonju, 26426 South Korea; 8 Dental Biomaterials, Departamento de Odontología, Facultad de Ciencias de la Salud, University CEU-Cardenal Herrera, Valencia, Spain; 9 Tissue Engineering & Biophotonics, King’s College London Dental Institute (KCLDI), London, UK; 10 Department of Nanobiomedical Science & BK21 PLUS NBM Global Research Center for Regenerative Medicine Research Center, Dankook University, Cheonan, 31116 South Korea

## Abstract

Restoration of hard tissue in conjunction with adhesive is a globally challenging issue in medicine and dentistry. Common clinical therapies involving application of adhesive and substitute material for functional or anatomical recovery are still suboptimal. Biomaterials with bioactivity and inhibitory effects of enzyme-mediated adhesive degradation can render a solution to this. Here, we designed a novel copper-doped bioactive glass nanoparticles (CuBGn) to offer multifunction: metalloproteinases (MMP) deactivation and remineralization and incorporated the CuBGn in resin-dentin adhesive systems, which showed most common failure of MMP mediated adhesive degradation among hard tissue adhesives, to evaluate proposed therapeutic effects. A sol-gel derived bioactive glass nanoparticles doping 10 wt% of Cu (Cu-BGn) for releasing Cu ions, which were well-known MMP deactivator, were successfully created and included in light-curing dental adhesive (DA), a filler-free co-monomer resin blend, at different concentrations (up to 2 wt%). These therapeutic adhesives (CuBGn-DA) showed enhanced (a)cellular bioactivity, cytocompatibility, microtensile bond strength and MMP deactivation-ability. In conclusion, the incorporation of Cu ions releasing nano-bioactive glass demonstrated multifunctional properties at the resin-dentin interface; MMP deactivation and remineralization, representing a suitable strategy to extend the longevity of adhesive-hard tissue (i.e. resin-dentin) interfaces.

## Introduction

Enzymatic degradation and hydrolysis represent the main mechanisms responsible for the relatively “short-term” longevity of resin based adhesive-hard tissue interfaces created with simplified adhesive systems^1–3^. Therefore, preserving the structural integrity of such interfaces is a key factor for successful long-term bonding/sealing to hard tissue^4,5^. Inactivation of proteolytic enzymes (e.g. matrix metalloproteinases (MMPs)) within the adhesive-hard tissue hybrid layer is one of the main strategies to increase the longevity of the adhesive-hard tissue interface^6^. Indeed, when bone or dentin is etched with acids before adhesive adjustment or bonded with self-etching adhesives, MMPs are exposed and activated; this phenomenon also occurs in presence of organic acids produced by inflammatory bacteria^7–9^.

The application of adhesives or pre-treatments doped with anti-MMPs on hard tissue have been widely investigated to reduce proteolytic enzymes, especially in dentin-adhesive interface due to its most common failure in clinical performance via MMP mediated enzymatic degradation^10–12^. In addition, the longevity of resin-dentin interfaces can also extended by using bioactive restorative approaches^13^. It has been advocated that enzymatic-mediated matrix degradation can be reduced through mineral fossilization of active endogenous proteases^11,14^. Indeed, the incorporation of bioactive glasses into resin-based materials has been demonstrated to induce remineralization and preservation of the resin-dentin interface due to their unique ability to release calcium (Ca^2+^) or phosphate (PO_4_^3−^)^15–17^.

One of the current trends in biomaterials research is the generation of nano-scaled mesoporous bioactive glasses (BGn) with enhanced surface-area/volume-ratio and greater ion-releasing properties, which tackle microsize-induced limitations of conventional melt-quench derived bioactive glasses as an additive in adhesive resin: increase in thickness and viscosity of adhesive layer and insufficient infiltration into dentinal tubules^18^. The bioactivity of such BGn could be potentiated by doping with specific functional and therapeutic ions, such as Sr, Ag, F, Fe, and Cu^19–21^. For instance, fluoride (F)-doped bioglasses incorporated into resin-based materials have been demonstrated to have greater remineralization properties and MMP inhibition ability compared to bioglass 45S5^16^. Among many therapeutic ions, copper (Cu^2+^) is considered a potent inhibitor of MMPs in human dentin^22,23^. However, whether Cu-doped BGn (CuBGn) capable of additional release of Cu^2+^ in addition to Ca^2+^ and Si^4+^ from BGn simultaneously may promote dentin remineralization and inhibit dentin-matrix degradation has not been investigated.

**Figure 1:**
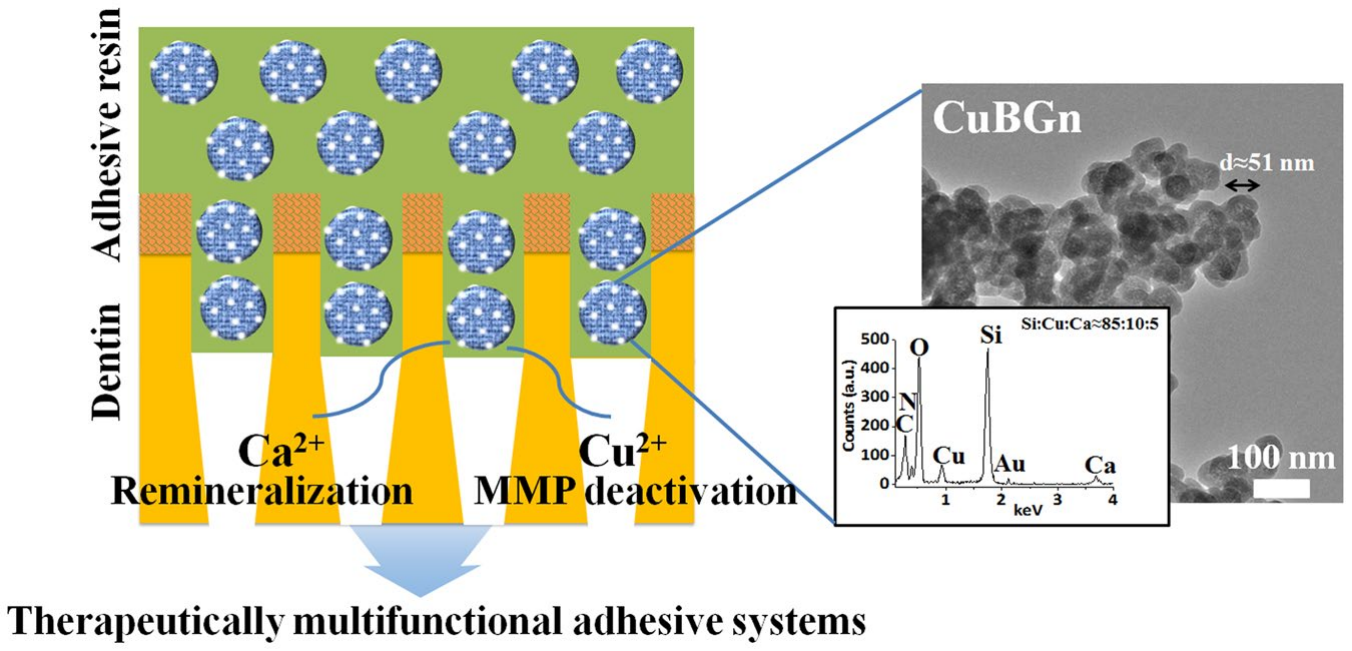
Therapeutically multifunctional adhesive systems using copper doped bioactive glass nanoparticles. Schematically illustrated the multifunctional roles of Ca and Cu ions delivered through nanoparticles (CuBGn) in adhesive resin. The co-delivery of Ca and Cu ions respectively accelerate remineralization and MMP deactivation in demineralized dentin, and ultimately extend the longevity of resin-dentin interfaces dentin regeneration. TEM images (right images) of CuBGn with EDS results (insert, n = 3) confirming the designated composition of nanoparticles.

Thus, the aim of this study was to generate and characterize a novel CuBGn and evaluate multifunctionally therapeutic adhesive systems incorporating CuBGn in terms of MMP inhibition from Cu ions and remineralization ability from Ca ions, along with the cytocompatibility and cellular-bioactivity (Fig. 1). The null hypothesis of this study states that MMP inhibition and remineralization ability of CuBGn incorporated adhesive systems do not differ significantly from those of adhesive systems without CuBGn.

## Materials and Methods

### Preparation of the Cu-doped nano-bioactive glass and experimental adhesives

Surface silanated Cu-free (BGn) and Cu-doped mesoporous bioactive glass nanoparticles (CuBGn) were prepared as previously described^20,24^. Morphology and elemental characterization was performed using transmission electron microscope (TEM, JEOL 7100; JEOL, Tokyo, Japan) and energy-dispersive X-ray spectroscopy (EDS, Inca 300; Oxford, Abingdon, UK), respectively. The surface area, pore size and pore volume of the tested bioglasses were calculated through an N_2_ adsorption-desorption analysis and Brunauer-Emmett-Teller (BET) equation^20^. The two bioactive glasses were silanized (+20–24 mV) using 3-Aminopropyltriethoxysilane^24^. The CuBGn bioglass was incorporated at different concentration (0.5 wt%, 1.0 wt% and 2 wt%) into a co-monomers resin blend (DA) consisting of 70 wt% bisphenol A diglycidyl ether dimethacrylate (Bis-GMA), 28.75 wt% 2-hydroxylethyl methacrylate (HEMA), 1 wt% ethyl N,N-dimethyl-4-aminobenzoate (EDMAB) and 0.25 wt% camphorquinone (CQ)^25^, in order to generate several light-curing experimental CuBGn-DA adhesives. DA served as a control filler-free adhesive. The Cu-free (BGn) was also incorporated (2 wt%) into the resin blend to create a further control experimental bioactive adhesive (BGn-DA) for comparisons. All chemicals were purchased from Sigma/Aldrich Chemical Co. and used as received, unless otherwise stated.

### Assessment of ion release, non-cellular bioactivity, water sorption and solubility

Three resin-disk specimens were prepared for each tested material using silicon molds (ϕ = 10 mm; h = 2 mm) and light-cured for 40 s using a LED curing system (Litex 695; Dentamerica Inc., Industry, CA, USA). Subsequently, the specimens were polished using SiC papers up to #1000-grit under continuous distilled water (DW) irrigation. Specimens were immersed in DW (3 cm^2^/ml) and supernatants were collected to evaluate the ions release (Ca, Si, Cu) up to 28 days (Fig. 1A); this was performed using inductively coupled plasma atomic emission spectroscopy (ICP-AES) (Optima 4300 DV; PerkinElmer, Waltham, MA, USA). Further specimens were created as previously described and incubated in simulated body fluid (SBF) for 28 days at 37 °C and pH 7.4. The surface of the specimens were analyzed before and after SBF immersion using scanning electron microscopy (SEM, Sigma 500; ZEISS, Oberkochen, Germany), X-ray diffraction (XRD, Ultima IV; Rigaku, Inc., Danvers, MA, USA, 2θ = 5–70°, 2° min^−1^), and FT-IR (Optima 4300DV; Perkin-Elmer) to evaluate the bioactivity of the adhesives doped with bioactive glasses^19,20^. A further five specimens (ϕ = 15 mm; h = 1.0 mm) were created for each tested material and assessed for water resorption and solubility^19^.

### Cytocompatibility and cellular bioactivity assay

Five resin-disk specimens (ϕ = 15 mm; h = 1.0 mm) were created for each tested material. Human dental pulp stem cells (hDPSCs) were extracted from third molars after approval from Dankook University Dental Hospital (H-1407/009/004) and cytocompatibility test was performed according to ISO standard procedures^26^ and processed as recently described^27^. Briefly, 100 µl of 1 × 10^5^ cells/mL were cultured in each well of a 96-well plate (SPL LifeSciences, Pocheon, Gyeonggi-do, Korea) with supplemented media, composing of alpha-minimum essential medium (Gibco, Waltham, MA, USA), 10% fetal bovine serum (Gibco), 1% penicillin/streptomycin (Invitrogen), 2 mM GlutaMAX (Gibco), and 0.1 mM L-ascorbic acid, in a humidified atmosphere of 5% CO_2_ at 37 °C for 24 h. After being washed with phosphate buffered saline (PBS, 200 µl), the cells were co-cultured with 50 µl of 2X supplemented media and 50 µl of extract or serially diluted extract by DW for another 24 h. The percentages of the final concentrations of extract in the culture media were 50%, 25%, 12.5% and a mixture of 50 µl of DW with 50 µl-supplemented media was used as the control (0%). Extracts (3 cm^2^/ml) were obtained after 24 h of DW immersion^28^ and filtered (0.2 µm). Cell viability was assessed with the water soluble tetrazolium (WST) assay (EA-Cytox; Daeil Lab, Seoul, Korea) was used according to the manufacturer’s protocol (n = 5). Following the addition of WST reagent at the ratio of 1:10 in supplemented media, after 2 h of incubation in 5% CO_2_ in a humidified atmosphere at 37 °C, the optical density (OD) was measured by reading the absorbance at 450 nm in a microplate reader against a blank column^29^. Cell proliferation was calculated as a ratio of OD from experimental value to control (0%), both subtracted by blank control^30^.

### qPCR methodology

Quantitative real-time PCR (qPCR) was performed with hDPSCS cultured with non-cytotoxic 25% extract under DM after 21 days, and relative odontogenic gene markers, such as collagen type 1 alpha (Col1a), dentin matrix protein 1 (DMP1), dentin sialophosphoprotein (DSPP), and osteocalcin (OCN)^19,20^ were determined compared to housekeeping gene (Glyceraldehyde 3-phosphate dehydrogenase, GAPDH, n = 3). Total RNA was extracted from the MSCs using Ribospin (GeneAll, Seoul, Korea), and 1 µg of RNA was reverse-transcribed to cDNA with oligo-dT (Venlo, Netherlands, Qiagen), a pre-mixture (AccuPower RT PreMix, Bioneer, Korea), and a 2720 Thermal Cycler (Applied Biosystems, Foster City, CA, USA). Quantitative mRNA expression level was measured using q PCR experiments with a SYBR Green (Applied Biosystems) and StepOnePlus machine (Applied Biosystems) according to the manufacturer’s instructions.

qPCR was performed using the following primer sequences: GAPDH, forward 5′-ACATCAAGAAGGTGGTGAAG-3′ & reverse 5′-AAAT GAGCTTGACAAAGTGG-3′; COL1a, forward 5′-AAGTCTTCTGCAACATGGAG-3′ & reverse 5′-TACTCGAACTGGAATCCATC-3′; DMP1, forward 5′-CCCTTGGAGAGCAGTGAGTC-3′ & reverse 5′-CTCCTTTTCCTGTGCTCCTG-3′; DSPP, forward 5′-GAAGATGCTGG CCTGGATAA-3′ & reverse 5′-TCTTCTTTCCCATGGTCCTG-3′; and OCN, forward 5′-AGCAAAGGTGCAGCCTTTGT-3′ & reverse 5′-GCGCCTGGGTCTCTTCACT-3′.

### Alizarin red staining methodology

Alizarin red staining (ARS) staining was performed to determine the mineralization ability of the noncytotoxic diluted extract (25%) using 1.2 ml of 10^5^/ml hDPSCs in each well of a 12-well plate (n = 3)^19,31,32^. Every 3 days, the media was replaced with fresh diluted extract (25%) conditioned odontogenic differentiation media (DM), consisting of ascorbic acid (50 mg/ml), b-glycerophosphate (10 mM), and dexamethasone (100 nM) in addition to SM; DW (25%) was added to the DM as a positive control, and DW (25%) was added to the supplemented media as a negative control. After 28 days of incubation, the cells were rinsed, fixed with 10% formaldehyde for 30 min, and stained with 40 mM alizarin red S (pH 4.2) for 30 min. After staining, the morphology was observed using light microscopy (Olympus lX71; Shinjuku, Tokyo, Japan). Quantitative analysis was performed after the addition of 10% cetylpyridinium chloride (Sigma Aldrich) in 10 mM sodium phosphate (pH 7.0) for de-staining. The concentration of ARS was determined by an absorbance measurement at 562 nm on a microplate reader (SpectraMax M2e; Molecular devices, Sunnyvale, CA, USA).

### Confocal microscope analysis in hybrid layer

Nanoparticles, DA, and dentin were processed for fluorescence staining as previously described in the literature^33–35^. Nanoparticles were incubated in a 7-Amino-4-methyl-3-coumarinylacetic acid solution (10 mg/ml; λex 350 nm; λem 433 nm) for 12 h^33,34^. Subsequently, CuBGn (2 wt%) and BGn (2 wt%) were incorporated into the DA, which was previously doped with fluorescein isothiocyanate isomer (1 mg/ml; λex 492 nm; λem 518 nm), in order to generate two experimental adhesives for confocal microscopy evaluation; DA was used as filler-free control resin. Human third molars recently extracted for surgical reasons under a protocol approved by the ethics committee (IRB number H-1407/009/004) were stored in deionized water (pH 7.4) at 4 °C no longer than 3 months^27^.

Middle dentin specimens (DS) were obtained by removing the roots 2 mm below cemento–enamel junction (CEJ) and with a parallel cut at 2 mm above CEJ using a slow-speed water-cooled diamond saw (Isomet; Buehler, Lake Bluff, USA). The dentin surface was wet-polished with 600-grit SiC papers for 1 min. Each DS was etched with 37% phosphoric acid for 15 s (3 M Scotchbond; 3 M ESPE, Maplewood, MN, USA), rinsed with DW (20 s) and DA excess removed with a microbrush. The CuBGn-DA and BGn-DA were solvated in acetone (1:1 vol%) and applied for 20 s onto DS, air-dried for 5 s and light cured for 20 s (Litex 695). A bulk-fill composite (Venus; Heraeus Kulzer, Hanau, Germany) was used for build-ups along with latex molds (6 × 6 × 4 mm), and finally light-cured (20 s). The specimens were cut in slabs (1 mm^2^) and immersed in a calcium-staining dye for 24 h (0.5 wt% xylenol orange; λmax 580 nm) as described by^35^. Fluorescence images were taken using confocal microscope (LSM 700; Carl Zeiss, Oberkochen, Germany) in order to assess the ability of CuBGn-DA to diffuse into the etched-dentin and form the hybrid layer.

### MMP Inhibition, microtensile bond-strength and remineralization tests

To determine the total MMP activity from matrix-bound MMPs, ten human dentin beams per each tested materials (2 × 1 × 6 mm) were etched using 10% phosphoric acid for 12 h at 25 °C (10 rpm). Absence of residual minerals was confirmed using digital radiography. The beams were immersed in 100 µl of acetone solution (50%) or DW (control) or in experimental adhesives-acetone mixture (1:1 vol%) for 5 min under continuous agitation. The total MMP activity was determined as previous methodology using a generic MMP assay (AnaSpec Inc., Fremont, CA, USA) for 1 h at 25 °C^36^.

Three further teeth were prepared and bonded as describe above, but using no fluorescent dyes. Dentin beams (1 mm^2^) were cut after 24 h of DW immersion using a slow speed diamond saw (ISOMET). Twenty beams were submitted to microtensile bond strength tester (Bisco, Schaumburg, IL, USA) at a speed of 1 mm/min^37,38^, while three beams from each group were first aged in NaOCl 10% for 1 h and finally incubated in SBF solution (3 cm^2^/ml; replaced every other day) for 14 days in order to investigate the remineralization ability induced by the (Cu)BGn incorporated hybrid layer *per se*, using SEM (Sigma 500) and EDS (Ultradry; Thermo Fisher Scientific, Waltham, MA, USA)^27^.

### Statistical analysis

Data were expressed as means ± S.D. All experiments were independently carried out in triplicate to confirm reproducibility. The data were analyzed using one-way analysis of variance (ANOVA), followed by Tukey’s post hoc test. The level of significance was p < 0.05. Fisher’s test was used to assess the significant difference in failure modes between experimental groups.

## Results

### Characterization of CuBGn and BGn

Nanospheres of CuBGn as well as BGn presented a size of approximately 50~85 nm and a worm-like mesoporous structure (Figs 1 and S1A). The EDS showed that the weight ratio of Si:Cu:Ca (wt%) in CuBGn was 85.8 ± 0.8: 9.6 ± 0.8: 4.6 ± 1.1. The BGn had a weight ratio of 85.5 ± 1.3 for Si and 14.5 ± 1.3 for Ca (Fig. S1A). Particle size (50.6 ± 4.9), surface area (34.1 m^2^/g), pore volume (0.060 ± 0.003 cm^3^/g), and average pore size (~7.0 nm) were recorded for surface of CuBGn, while, BGn showed a relatively higher particle size (85.4 ± 7.2), surface area (39.7 m^2^/g), pore volume (0.084 ± 0.004 cm^3^/g), and average pore size (~8.8 nm) (Fig. S1B). The charge of the surfaces, as measured by zeta-potential, was positive due to surface amination (CuBGn: 24.2 ± 0.94 mV; BGn: 20.2 ± 0.71 mV).

### Ion-release and non-cellular bioactivity of CuBGn-doped adhesives

**Figure 2:**
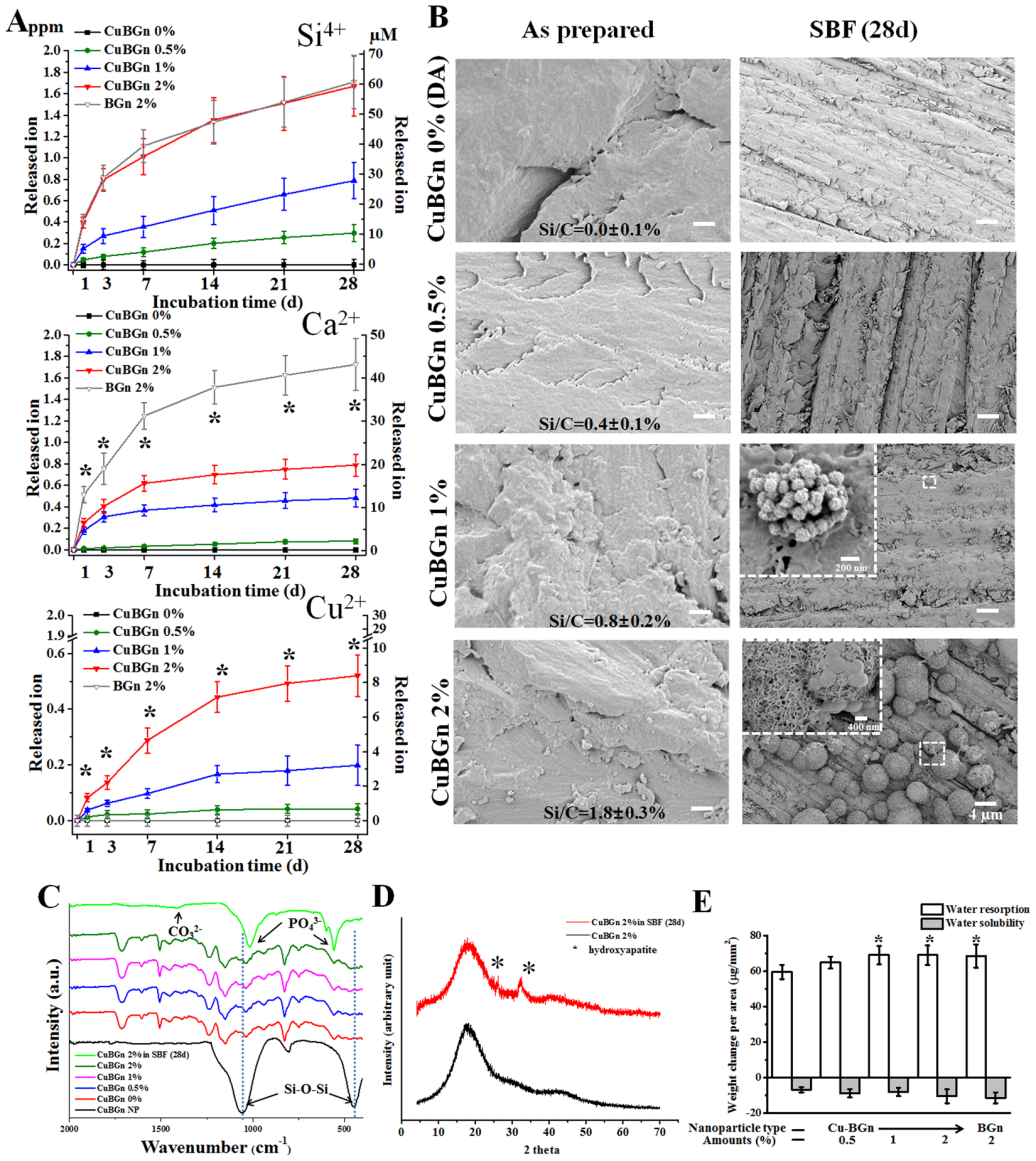
Release of ions, non-cellular bioactivity and water resorption/solubility. (**A**) Ion release from set (Cu)BGn-DA specimens as assayed by ICP-AES during incubation at 37 °C while shaking at 120 rpm in DW (3 cm^2^/mL); released amounts shown in [ppm] and [µM]; significant difference noted between CuBGn and BGn (*p < 0.05, n = 3). (**B**–**D**) Non-cellular bioactivity after immersion in 37 °C SBF for 28 days as visualized or determined using SEM, FTIR and XRD. (**B**) CuBGn-DA 1% and 2% immersed in SBF exhibited hydroxyapatite (HA)-like precipitation on the surface under SEM. Poorly crystalized HA was confirmed by (**C**) FTIR and (**D**) XRD. (**E**) Water resorption and solubility were measured according to ISO 4049 (n = 5).

The resins doped with CuBGn showed important release of Si, Ca, and Cu ions; no Cu ions were detected with the BGn-DA (Fig. 2A). Si ion release from CuBGn-DA(2%) and BGnDA(2%) was similar in terms of total release amount (~1.7 ppm). However, Ca ions were released more slowly from CuBGn-DA(2%) than BGn-DA(2%), and the total amount released from CuBGn-DA(2%) was approximately half that released from BGn-DA(2%), (~0.8 ppm vs. ~1.7 ppm), respectively. Up to 28 days, total Cu ion release was ~0.5 ppm for CuBGn-DA(2%) with a gradual decrease in release pattern during the test period (Fig. 2A).

The surface morphology of the experimental resins after a 28-day incubation period in SBF showed clear HA-like crystallites in BGn-DA(1%) and BGn-DA(2%); However, CuBGn-DA(2%) showed the greatest presence of HA (Fig. 2B). EDS confirmed the successful incorporation of CuBGn in DA showing a specific Si/C ratio (~0, ~0.4, ~0.8 and ~1.8%) before SBF immersion. FT-IR peaks representing CuBGn (Si-O-Si) and resin monomers were observed in CuBGn-DA 0.5, 1, and 2%, and depending on the amounts of CuBGn incorporated into the resin, the intensity of Si-O-Si peaks was more evident (Fig. 2C). Peaks of carbonate and phosphate consisting of HA were clearly detected only in CuBGn-DA(2%) after 28-day of SBF immersion (Fig. 2C). The XRD pattern confirmed the formation of HA in CuBGn-DA(2%) after 28-day of SBF incubation (Fig. 2D). XRD patterns of CuBGn-DA(2%) prior to SBF immersion only exhibited a broad amorphous peak at 2Ɵ = 10–40°.

All the experimental resins containing bioactive fillers exhibited comparable water resorption (65–70 µg/mm^2^) and solubility (8–13 µg/mm^2^) to the control resin (60 µg/mm^2^ of resorption; 6.8 µg/mm^2^ of solubility µg/mm^2^).

### Cytocompatibility and cellular bioactivity assay

**Figure 3:**
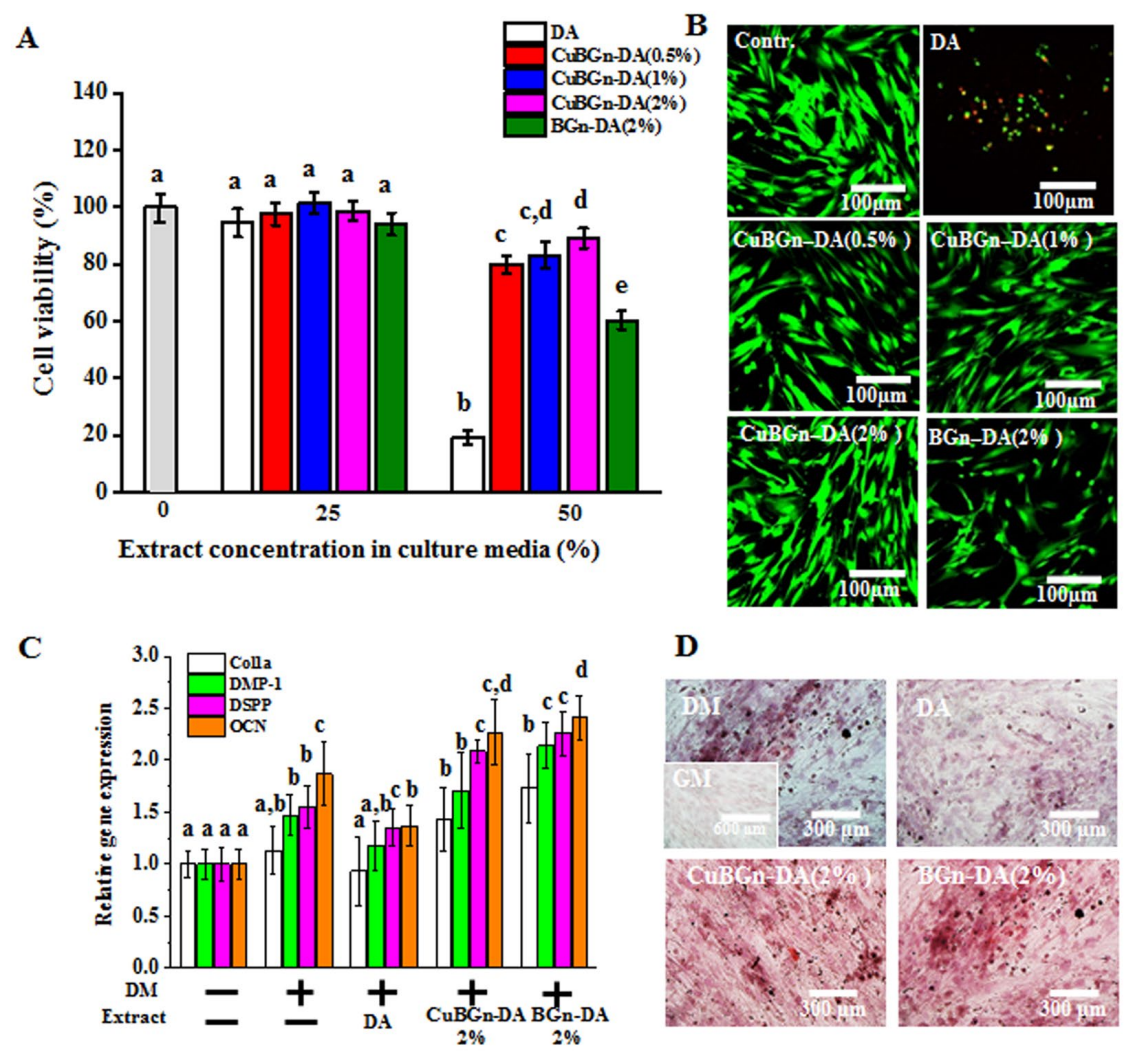
Cytocompatibility and cellular bioactivity in hDPSC differentiation. Cytocompatibility against hDPSCs was measured using WST (**A**) and a live-dead (**B**) assay, which revealed a significant increase in cytocompatibility in (Cu)BGn-DA compared to that in the DA control (n = 5). Cellular bioactivity in hDPSCs differentiation was analyzed using (**C**) qPCR (21 d, n = 3) and (**D**) alizarin red staining (28 d, n = 3). Significant increases in odontogenic differentiation markers, such as Col1, DMP1, DSPP and OCN, were detected in extracts from (Cu)BGn-DA compared to their expression in DA. A higher degree of mineralization (red) was detected in (Cu)BGn-DA than in DA. Different letters indicate a significant difference between groups at a level of 0.05. DM: differentiation media. GM: growth media.

Cell viability (hDPSCs) with 50% extract was significantly enhanced: CuBGn: CuBGn-DA(2%) ≥ CuBGn-DA(1%) ≥ CuBGn-DA(0.5%) > BGn-DA(2%) > filler-free resin (Fig. 3A, p < 0.05). This was also visualized in live and dead images (Fig. 3B). The 25% extract treatment produced no significant difference in cell viability compared to the control. Moreover, the following order of gene expression was observed in descending order: BGn-DA(2%), CuBGn-DA(2%), DA, DM, and GM (Fig. 3C). A significant increase in all types of gene expression in CuBGn-Da(2%) and BGn-DA(2%) compared to that in DA was observed (p < 0.05). Alazarin red staining revealed significantly higher staining in CuBGn-DA(2%)and BGn-DA(2%) than in the controls (Fig. 3D).

### MMP Inhibition, microtensile bond-strength and remineralization tests

**Figure 4:**
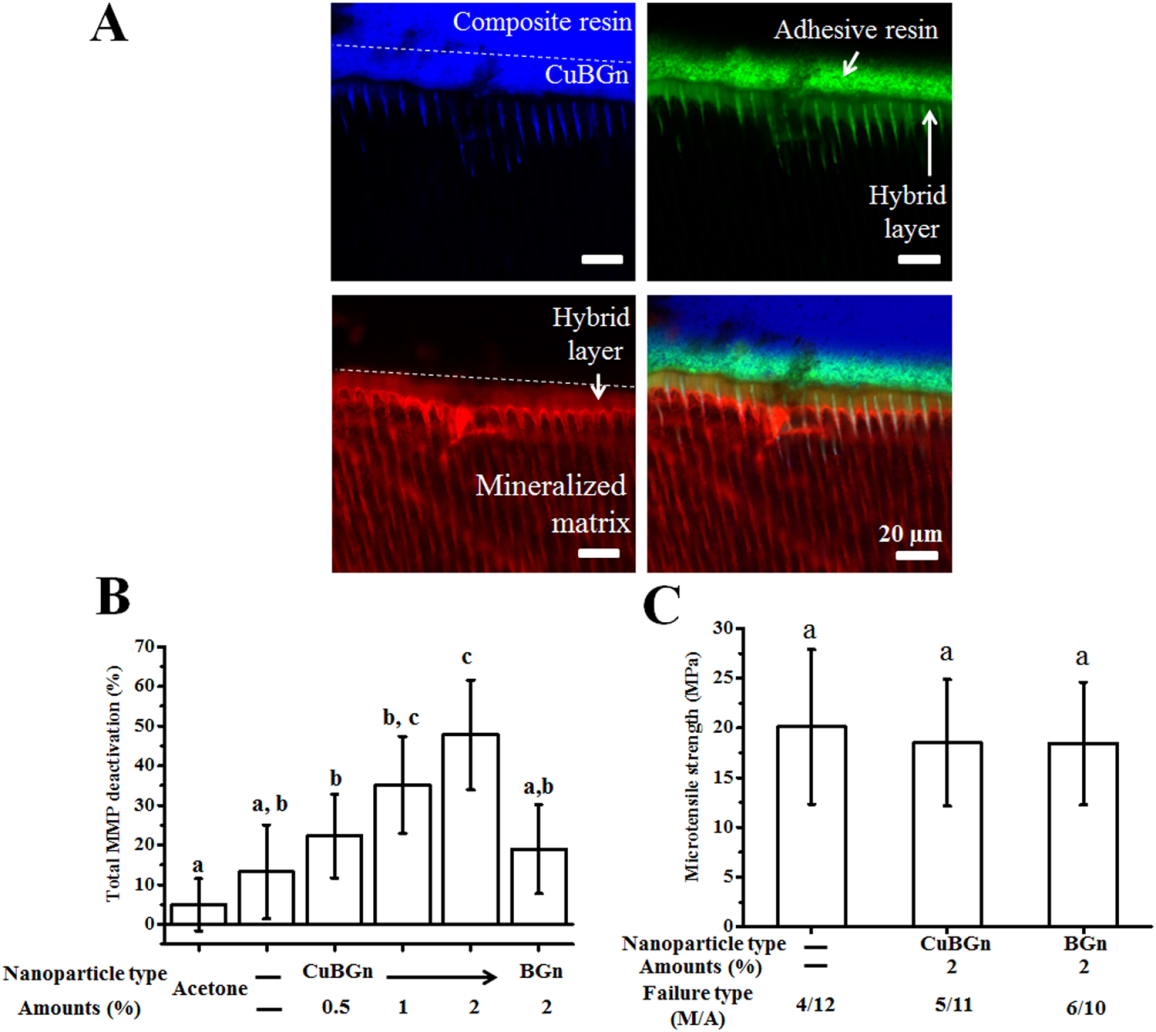
Endogenous MMP deactivation and microtensile strength after CuBGn@DA application to dentin. (**A**) Fluorescent images reveal the successful application of CuBGn (blue)-incorporated DA (green) on dentin (mineralized matrix, red). Auto-fluorescence of composite resin was also shown as blue (**B**) Endogenous MMP activity was significantly decreased in CuBGn-DA compared to that in the controls (BGn-DA, DA, and acetone (solvent of DA)) after application of DA to dentin etched with 37% phosphoric acid. (**C**) Microtensile strength was not compromised after the incorporation of nanoparticles (n = 20). Different letters indicate significant differences between groups at a level of 0.05.

Blue dye-incorporated CuBGn filler was homogenously observed in DA (green), which infiltrated into the mineralized dentin matrix (red); diffusion of CuBGn within the hybrid layer and into the dentinal tubules was clearly evident (Fig. 4A). MMP activity assessment showed that acid-etched dentin treated with CuBGn-DA(2%) significantly reduced the total MMP activity compared to the control and to BGn-DA(2%) (Fig. 4B, p < 0.05). However, no significant difference in bond strength was observed between the two experimental adhesives and the control DA (Fig. 4C, p > 0.05), as well as no significant difference in failure modes; the failure mode in all group was mainly mixed and adhesive (p > 0.05).

**Figure 5:**
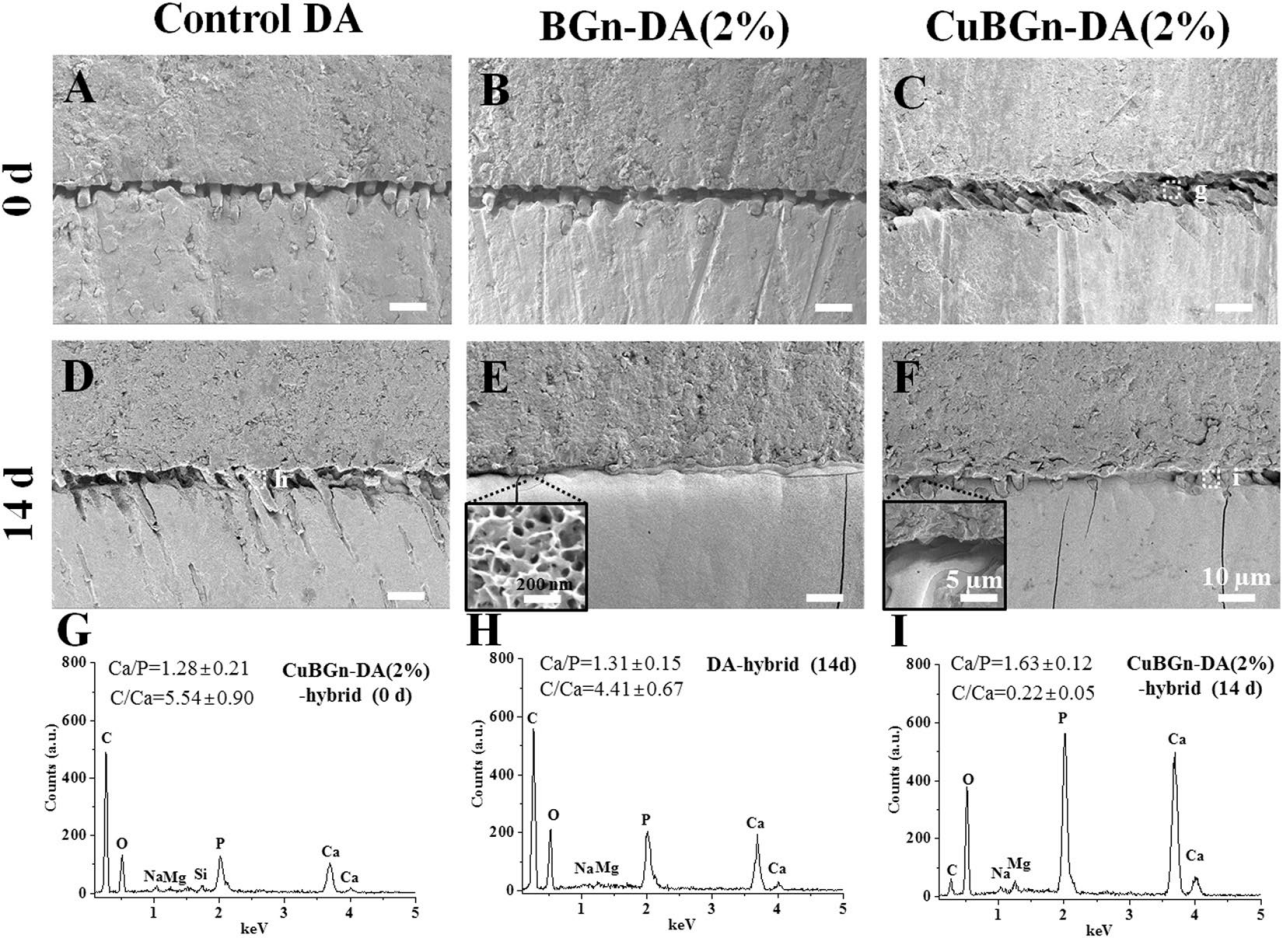
Non-cellular bioactivity in (Cu)BGn-DA applied dentin after SBF immersion. (Cu)BGn-DA was applied to dentin, and bulk-fill resin was stacked up to create a complex of composite resin-DA-dentin structure. Dentin-composite resin sticks (1 mm^2^) were created and treated with 10% NaOCl for 1 h for chemical aging to remove denuded collagen for inhibiting spontaneous HA precipitation and immersed in SBF (3 cm^2^/mL) for 14 days. The adhesive interface, including around the spot, was characterized using (**A**–**F**) SEM and (**G**–**I**) EDS before and after SBF immersion for 14 days. Dot white box in C and F indicating spot for EDS analysis. Elemental ratio was obtained from three different analyses and representative EDS graph was shown. HA like precipitation (insert in **E** and **F**) was observed and determined by EDS analysis in CuBGn-DA(2%) incorporated hybrid layer after 14 days of SBF incubation but not in DA incorporated hybrid layer.

After NaOCl challenging, it was detected that CuBGn-DA(2%) induced comparable remineralization within the hybrid layer to that of BGn-DA(2%) after 14 days of incubation in SBF (Fig. 5). HA-like crystals were revealed typically within the hybrid layer (Fig. 5E,F). EDS analysis revealed that the Ca:P ratio (at%) of HA-like crystals in CuBGnDA(2%) within the resin-dentin interface changed from before (1.28 ± 0.21, Fig. 5G) to after (1.63 ± 0.12, Fig. 5I) SBF incubation; this latter was comparable to that of HA (~1.67). Conversely, the resin-dentin interfaces created with the filler-free control DA showed no HA formation. The C:Ca ratio (%) within the hybrid layer created with CuBGn-DA(2%) before SBF immersion (5.54 ± 0.90) was similar to that created with the control DA after SBF immersion (4.41 ± 0.67). While, CuBGn-DA(2%) showed a significant lower C:Ca ratio (%) value (0.22 ± 0.05) after prolonged SBF immersion.

## Discussion

With the limitations of this study in mind, according to the above results, the null hypothesis stating MMP inhibition and remineralization ability of CuBGn incorporated adhesive systems do not differ significantly from those of adhesive systems without CuBGn was rejected because CuBGn-DA showed significant increase of (bio)remineralization as well as MMP deactivation compared to DA.

First, this study fabricated BGn/CuBGn and performed their post treatment for being incorporated in polymer based DA. This study has demonstrated successfully silanization (+20~24 mV) and incorporation of mesoporous (average: 7–9 nm) bioactive glass nanoparticles (ϕ ≈ 50–85 nm) with relatively large surface areas (34–40 m^2^/g) into experimental resin adhesives. These nano-scaled mesoporous particles have great merits as an additive in adhesive resin compared to conventional microsized melt-quench derived bioactive glasses in terms of their homogeneous dispersion in adhesive resin with little impact to wettability and thickness of adhesive layer and capacity of dentinal tubules (d = 0.9~2.5 µm) infiltration^18^. Moreover, a sustained release of Ca and Si ions, therapeutic ions able to induce remineralization, was attained over 28 days due to the slow degradation rate of the BGn and CuBGn nanoparticles in the experimental adhesives; the total amount of ions released from such therapeutic adhesives depended on the incorporation amounts (up to 2%). Indeed, CuBGn-DA was able to release up to 0.5 ppm Cu ions over a period of 28 days, which was in the effective range for MMP deactivation^39^. CuBGn2%-DA and BGn2%-DA exhibited bioactivity under SBF solution immersion. An abundance of poorly crystallized HA was precipitated on the surface; this was also confirmed by FTIR and XRD (Fig. 2B–D). However, concerns about excessive degradation of bioactive glasses in DA may be excluded in these materials, as the results of water solubility obtained in this study showed similar values to the control DA. Conversely, the negative values of water solubility and significant higher water sorption obtained with the tested experimental DAs containing bioactive glasses after DW incubation (Fig. 2E) may support their ability to evoke bioactive processes, as previously explained by^40^.

The cytocompatibility of the experimental adhesives doped with the tested BGn was significantly improved compared to that of the control DA. This was likely due to the catalytic activity of released metal ions (e.g. Cu ions) and to the lower monomer content^41^. Indeed, when the hDPSCs were cultured in odontogenic differentiation media with a non-cytotoxic concentration (25%) of extract up to 28 days, enhanced mRNA gene expression of odontogenic markers (COL1A, DMP-1, DSPP and OCN) was observed. Evident biomineralization was detected in particular with the BGn-DA(2%) and CuBGn-DA(2%) due to the presence of therapeutic ions, such as Ca and Si ions released from within the tested materials^27^. We hypothesize that such biological effects might induce formation of tertiary dentin or osteodentin structures along the walls of the pulp chamber.

When the resin-dentin interfaces created with the experimental FITC (green)-incorporated and fluorescent (blue) nano-bioglasses were analyzed using a confocal microscope, it was possible to observe an homogeneous distribution of the adhesive; this indicates the potential for therapeutic adhesive diffusion into demineralized dentin and the creation of a typical hybrid layer - usually observed in etch-and-rinse adhesives^42^. The analysis of the dentin-derived endogenous total MMP activity showed that CuBGn-DA(2%) significantly increased MMP deactivation compared to that of the other tested groups as well as in the control group containing acetone only. These results were likely due to the release of Cu ions, which have a strong inhibition ability on dentin MMPs^22,23^. However, this study is the first scientific report to demonstrate ‘MMP-deactivation via (Cu^2+^) ions releasable from a Cu-doped nano-bioglass incorporated in an adhesive system. Moreover, this study demonstrated that MMP deactivation was related to the amount of CuBGn incorporated into the DA; the higher the presence of CuBGn (up to 2%), the greater the MMP inhibition. Nevertheless, a maximum concentration of 2% of CuBGn was chosen in order to avoid an excessive increase of viscosity of the experimental adhesives as per the large volume/weight ratio of nanoparticles used in this study.

It is anticipated that the dentin-adhesive bonding ability can be compromised maximally when the experimental adhesives incorporate the highest amount of nanoparticles. Therefore, two representative groups [CuBGn-DA(2%) and BGn-DA(2%)] were selected as experimental groups. The microtensile bond strength testing was performed after chemical aging in a 10% NaOCl treatment for 1 h instead of long-term aging study, inducing unbound (adhesive free) collagen degradation within the hybrid layer and producing similar aging effects on microtensile specimens as when specimens were stored in 6 months of DW incubation^37^. The results obtained in this study revealed no significant interference of the bioglass nanoparticles with the bond strength and mode of failure compared to the control DA. Previous studies also revealed that the incorporation of therapeutic nanoparticles, such as silver (0.1%), amorphous calcium phosphate (20%), or sepiolite (~2%), into two-step etch-and-rinse DA had no negative impact on shear bond and microtensile strength to dentin^43,44^. However, to precisely confirm MMP deactivation effects in terms of dentin-adhesive bonding ability, direct long-term microtensile experiments using DW, PBS or clinically relevant enzymatic environment are necessary, which will be further studied with in depth MMP deactivation mechanism study from released Cu ions.

The ability of the experimental adhesive to remineralize the gap between the resin and the ‘collagen-degraded’ dentin created by the chemical aging dentin-DA stick was investigated after immersion in SBF solution for 14 days. The results of this study showed that (Cu)BGn-DA was able to remineralize the gap via HA-like precipitation. HA-like precipitation was confirmed by EDS analysis, which showed similar Ca/P atomic ratio (~1.63) to that of reference HA (1.67), as well as much decreased C/Ca atomic ratio (~0.2) compared to that observed in the experimental resin before SBF immersion (~4.4). We believe that this outcome was due to high amount of calcium and phosphates and Si ions diffusing from the CuBGn-DA 2% within the hybrid layer.

## Conclusion

In conclusion, The results showed that the CuBGn-DA (2%) is able to release Ca, Si, and Cu ions, which induce remineralization at adhesive resin-dentin interface and reduce the MMP-degradation activity in demineralized dentin without affecting the bonding performance. Such multifunctionally innovative adhesive systems can also activate and stimulate cellular (bio)mineralization *in vitro*. The chemical and biological properties of CuBGn such as bioactivity, MMP inhibition, cytocompatibility, and enhanced differentiation, make the CuBGn-DA(2%) system a promising multi-purpose therapeutic adhesive for clinical application.

## Electronic supplementary material

Dataset 1

## References

[1] Pashley DH (2004). Collagen degradation by host-derived enzymes during aging. J Dent Res.

[2] Helling AL (2017). *In vitro* enzymatic degradation of tissue grafts and collagen biomaterials by matrix metalloproteinases: improving the collagenase assay. ACS Biomater Sci Eng.

[3] Aguda AH (2014). Structural basis of collagen fiber degradation by cathepsin K. Proc Natl Acad Sci USA.

[4] Frassetto A (2016). Mechanisms of degradation of the hybrid layer in adhesive dentistry and therapeutic agents to improve bond durability—A literature review. Dent Mater.

[5] Farrar DF (2012). Bone adhesives for trauma surgery: A review of challenges and developments. Int J Adhes Adhes.

[6] Palosaari H (2003). Expression profile of matrix metalloproteinases (MMPs) and tissue inhibitors of MMPs in mature human odontoblasts and pulp tissue. Eur J Oral Sci.

[7] Mazzoni A (2006). Reactivation of inactivated endogenous proteolytic activities in phosphoric acid-etched dentine by etch-and-rinse adhesives. Biomaterials.

[8] Parks WC, Wilson CL, Lopez-Boado YS (2004). Matrix metalloproteinases as modulators of inflammation and innate immunity. Nat Rev Immunol.

[9] Krane SM, Inada M (2008). Matrix metalloproteinases and bone. Bone.

[10] Altinci P (2016). NaF Inhibits matrix-bound cathepsin-mediated dentin matrix degradation. Caries Res.

[11] Mazzoni A (2017). Substantivity of carbodiimide inhibition on dentinal enzyme activity over time. J Dent Res.

[12] Montagner A, Sarkis-Onofre R, Pereira-Cenci T, Cenci M (2014). MMP inhibitors on dentin stability: a systematic review and meta-analysis. J Dent Res.

[13] Sauro S, Pashley DH (2016). Strategies to stabilise dentine-bonded interfaces through remineralising operative approaches – State of The Art. Int J Adhes Adhes.

[14] Tezvergil-Mutluay A (2014). Zoledronate and ion-releasing resins impair dentin collagen degradation. J Dent Res.

[15] Abuna G (2016). Bonding performance of experimental bioactive/biomimetic self-etch adhesives doped with calcium-phosphate fillers and biomimetic analogs of phosphoproteins. J Dent.

[16] Tezvergil-Mutluay A (2017). Effects of composites containing bioactive glasses on demineralized dentin. J Dent Res.

[17] Sauro S, Osorio R, Watson TF, Toledano M (2012). Therapeutic effects of novel resin bonding systems containing bioactive glasses on mineral-depleted areas within the bonded-dentine interface. J Mater Sci Mater Med.

[18] Misra SK (2008). Comparison of nanoscale and microscale bioactive glass on the properties of P(3HB)/Bioglass® composites. Biomaterials.

[19] Lee J-H (2017). Drug/ion co-delivery multi-functional nanocarrier to regenerate infected tissue defect. Biomaterials.

[20] Lee J-H, Mandakhbayar N, El-Fiqi A, Kim H-W (2017). Intracellular co-delivery of Sr ion and phenamil drug through mesoporous bioglass nanocarriers synergizes BMP signaling and tissue mineralization. Acta Biomater.

[21] Saravanan S, Selvamurugan N (2016). Bioactive mesoporous wollastonite particles for bone tissue engineering. J Tissue Eng.

[22] de Souza AP, Gerlach RF, Line SRP (2000). Inhibition of human gingival gelatinases (MMP-2 and MMP-9) by metal salts. Dent Mater.

[23] Renne, W. G. *et al*. Novel bacteriostatic and anti-collagenolytic dental materials through the incorporation of polyacrylic acid modified CuQ nanoparticles, Vol. US20140037705 A1 (Musc Foundation For Research Development, 2014).

[24] Lee J-H, Kang M-S, Mahapatra C, Kim H-W (2016). Effect of aminated mesoporous bioactive glass nanoparticles on the differentiation of dental pulp stem cells. PLoS One.

[25] Kim YK (2010). Mineralisation of reconstituted collagen using polyvinylphosphonic acid/polyacrylic acid templating matrix protein analogues in the presence of calcium, phosphate and hydroxyl ions. Biomaterials.

[26] Roger P, Delettre J, Bouix M, Béal C (2011). Characterization of Streptococcus salivarius growth and maintenance in artificial saliva. J Appl Microbiol.

[27] Jun S-K, Lee J-H, Lee H-H (2017). The biomineralization of a bioactive glass-incorporated light-curable pulp capping material using human dental pulp stem cells. Biomed Res Int.

[28] Yu J (2007). Induced pluripotent stem cell lines derived from human somatic cells. Science.

[29] Jo J-K (2017). Rechargeable microbial anti-adhesive polymethyl methacrylate incorporating silver sulfadiazine-loaded mesoporous silica nanocarriers. Dent Mater.

[30] Jun S-K (2017). Biological effects of provisional resin materials on human dental pulp stem cells. Oper Dent.

[31] Castillo Diaz LA (2016). Osteogenic differentiation of human mesenchymal stem cells promotes mineralization within a biodegradable peptide hydrogel. J Tissue Eng.

[32] LoGuidice A, Houlihan A, Deans R (2016). Multipotent adult progenitor cells on an allograft scaffold facilitate the bone repair process. J Tissue Eng.

[33] Hass V (2016). Collagen cross-linkers on dentin bonding: Stability of the adhesive interfaces, degree of conversion of the adhesive, cytotoxicity and *in situ* MMP inhibition. Dent Mater.

[34] Klinger-Strobel M (2016). A blue fluorescent labeling technique utilizing micro- and nanoparticles for tracking in LIVE/DEAD(R) stained pathogenic biofilms of Staphylococcus aureus and Burkholderia cepacia. Int J Nanomedicine.

[35] Profeta AC (2013). Experimental etch-and-rinse adhesives doped with bioactive calcium silicate-based micro-fillers to generate therapeutic resin–dentin interfaces. Dent Mater.

[36] Sabatini C (2014). Inhibition of endogenous human dentin MMPs by Gluma. Dent Mater.

[37] Garbui BU, Botta SB, Reis AF, Matos AB (2012). Comparison of chemical aging and water immersion time on durability of resin-dentin interface produced by an etch-and-rinse adhesive. J Contemp Dent Pract.

[38] Jang J-H (2016). Comparative study of the dentin bond strength of a new universal adhesive. Dent Mater J.

[39] Guo H (2005). Effects of copper and zinc on the production of homocysteine-induced extracellular matrix metalloproteinase-2 in cultured rat vascular smooth muscle cells. Acta Cardiol.

[40] Sauro S (2013). Remineralisation properties of innovative light-curable resin-based dental materials containing bioactive micro-fillers. J Mater Chem B Mater Biol Med.

[41] Sugiyama K, Lee SW (1977). Effect of metal ion on the radical polymerization of methyl methacrylate. Macromol Chem Phys.

[42] Sauro S, Osorio R, Watson TF, Toledano M (2012). Assessment of the quality of resin–dentin bonded interfaces: An AFM nano-indentation, μTBS and confocal ultramorphology study. Dent Mater.

[43] Fallahzadeh F, Safarzadeh-Khosroshahi S, Atai M (2017). Dentin bonding agent with improved bond strength to dentin through incorporation of sepiolite nanoparticles. J Clin Exp Dent.

[44] Zhang K (2013). Effect of water-ageing on dentine bond strength and anti-biofilm activity of bonding agent containing new monomer dimethylaminododecyl methacrylate. J Dent.

