# Supplementary material for: Multi-functional nano-adhesive releasing therapeutic ions for MMP-deactivation and remineralization
Source: Sci Rep. 2018 Apr 4;8:5663. doi: 10.1038/s41598-018-23939-6 (PMC5884793; doi:10.1038/s41598-018-23939-6)
Supplement: Supplementary file 1 — Dataset 1 [file 41598_2018_23939_MOESM1_ESM.doc]

**Supplemental appendix**

**Appendix figure 1**

**
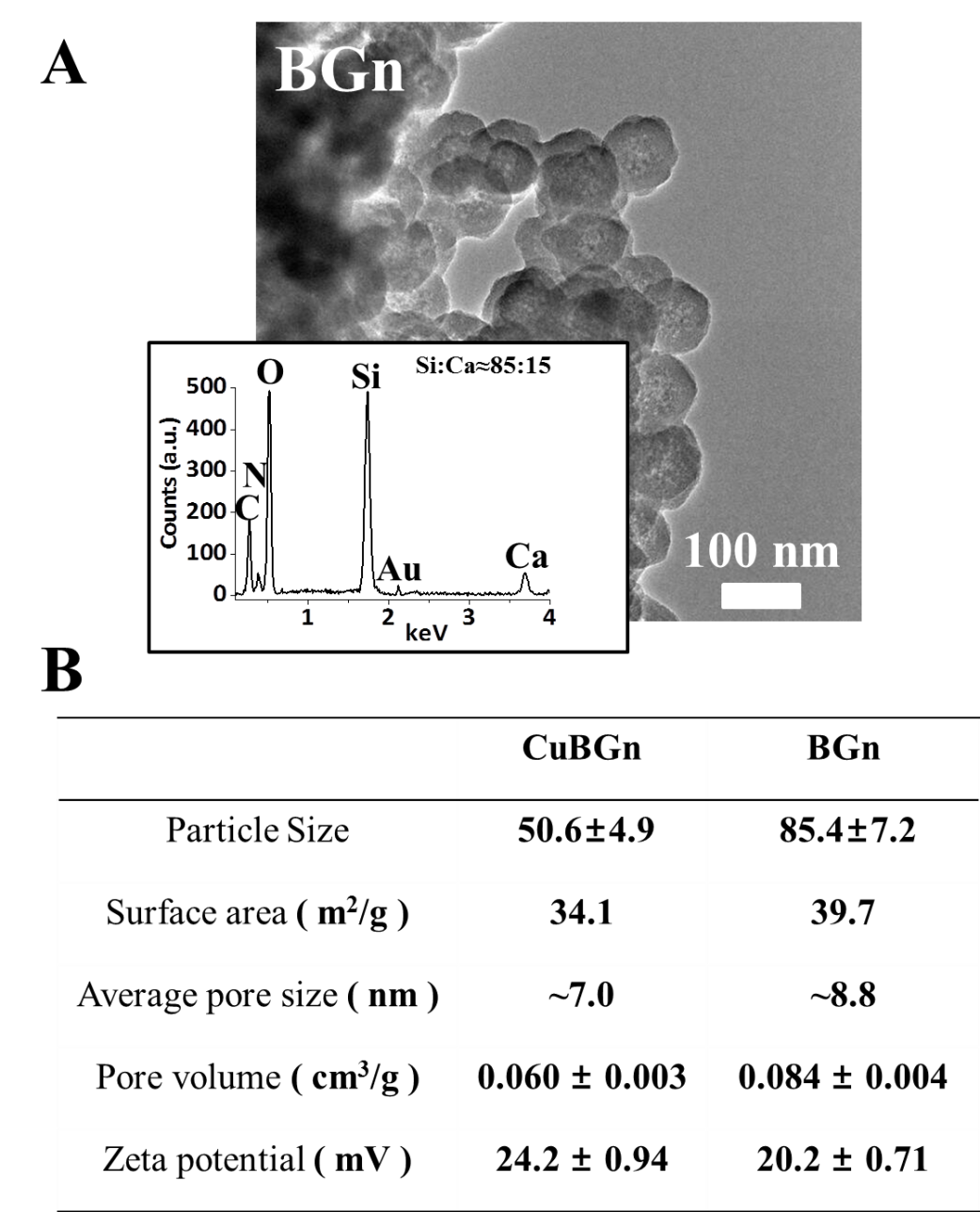
**

# Appendix figure 1. Characterization of nanoparticles. TEM images of (A) BGn with EDS results (insert, n=3) confirming the designated composition of nanoparticles. (B) Characteristics of CuBGn and BGn in terms of particle size, surface area, average pore size, pore volume, and zeta potential (n=3).
